# The HCMV terminase as an antiviral target: structural insights, inhibitory mechanisms, and the era of letermovir

**DOI:** 10.3389/fmicb.2026.1942629

**Published:** 2026-09-18

**Authors:** Guojun Chen, Yuqi Li, Jinrui Chen, Jiangqing Dong

**Affiliations:** 1 Hubei Shizhen Laboratory, Hubei University of Chinese Medicine, Wuhan, Hubei, China; 2 School of Pharmacy, Hubei University of Chinese Medicine, Wuhan, Hubei, China; 3 School of Basic Medical Sciences, Hubei University of Chinese Medicine, Wuhan, Hubei, China

**Keywords:** HCMV terminase, inhibitory mechanisms, letermovir, resistance mutations, structural organization

## Abstract

Human cytomegalovirus (HCMV) remains a major cause of morbidity and mortality in immunocompromised individuals and newborns worldwide. Current antiviral therapies mainly target the viral DNA polymerase, but their long-term clinical use is often constrained by toxicity and the emergence of drug-resistant strains. The approval of letermovir, the first antiviral targeting the viral terminase complex, has established viral genome packaging as a highly effective therapeutic strategy. The HCMV terminase complex is essential for viral DNA cleavage and encapsidation and lacks direct human homologs, making it an attractive target for selective antiviral intervention. Accumulating evidence indicates that letermovir may primarily target pUL56, as resistance mutations cluster predominantly within this subunit. This review summarizes current understanding of the interaction between letermovir and the HCMV terminase, focusing on structural organization, inhibitory mechanisms, resistance mutations, and emerging approaches for next-generation inhibitor development, and highlighting key unresolved questions that will guide future structure-based antiviral discovery targeting herpesvirus genome packaging machinery.

## 1 Introduction

Human cytomegalovirus (HCMV) is a member of the betaherpesvirus subfamily, infecting a large proportion of the global population and establishing lifelong persistence and periodic reactivation following primary infection (Fowler et al., 2022; Shang and Li, 2024; Xia et al., 2026). Although HCMV infection is usually asymptomatic in immunocompetent individuals, it poses severe risks in immunocompromised patients, including transplant recipients, AIDS patients, and individuals receiving immunosuppressive therapies (Griffiths and Reeves, 2021; Shang and Li, 2024). In addition, congenital HCMV infection remains one of the leading infectious causes of neurological defects and hearing loss in newborns (Rawlinson et al., 2017; Fowler and Boppana, 2018). These HCMV-related diseases have imposed a heavy medical burden worldwide (Modlin et al., 2004; Lawrence et al., 2024).

Current antiviral therapies against HCMV primarily target viral DNA replication (Bogner et al., 2021). Ganciclovir and valganciclovir inhibit viral DNA polymerase (pUL54) after first phosphorylation by the viral kinase pUL97 into the monophosphate form and subsequent phosphorylation by host kinases into active triphosphate form, whereas foscarnet and cidofovir directly interfere with polymerase function (Zarrouk et al., 2017; Bogner et al., 2021). Despite their efficacy, these drugs suffer from several major limitations. Long-term treatment frequently leads to toxic side effects, including bone marrow suppression and nephrotoxicity (Mercorelli et al., 2008; Hardinger and Brennan, 2024). Moreover, prolonged antiviral exposure promotes the emergence of resistant viral strains, particularly mutations within pUL54 and pUL97 (Carter et al., 2025; Alemán et al., 2026). This situation underscores an urgent need for novel anti-HCMV therapies.

The approval of letermovir represented a major breakthrough in anti-HCMV therapy because it introduced a mechanistically distinct antiviral strategy targeting viral DNA packaging rather than DNA synthesis (Goldner et al., 2011; Marty et al., 2017; Limaye et al., 2023). Letermovir specifically inhibits the viral terminase complex, a molecular machine responsible for cleavage and encapsidation of newly synthesized viral genomes into preformed capsids (Goldner et al., 2011; Gourin et al., 2025a). This discovery established the viral genome packaging pathway as a highly druggable vulnerability during HCMV replication. Importantly, the mechanism of letermovir differs completely from that of classical polymerase inhibitors, allowing effective suppression of strains resistant to ganciclovir or foscarnet (Goldner et al., 2011). These unique characteristics have generated tremendous interest in understanding how antiviral compounds interact with the terminase complex at molecular and structural levels.

The HCMV terminase complex is generally believed to consist of three major subunits: pUL56, pUL89, and pUL51 (Wang et al., 2012; Neuber et al., 2017, 2018). pUL56 is thought to function in packaging signal recognition in concatemeric viral DNA and ATP-associated regulation, while pUL89 contains catalytic nuclease motifs involved in DNA cleavage. pUL51 appears to act as an essential factor required for efficient complex assembly and cleavage-packaging of the HCMV genome. During viral replication, the terminase machinery recognizes concatemeric viral DNA, cleaves genome units at specific packaging sequences, and powers ATP-dependent translocation of viral DNA into capsids through the portal vertex (Dittmer et al., 2005). Despite increasing functional characterization of terminase components, many aspects of terminase biology remain poorly understood. A critical caveat is that much of our mechanistic understanding is extrapolated from the well-studied HSV-1 system; direct functional and structural evidence for the HCMV terminase is comparatively sparse, and the degree of mechanistic conservation across herpesviruses, while likely, remains an assumption that requires direct experimental validation. In particular, the precise structural basis of antiviral inhibition remains elusive. Although resistance mutations strongly implicate pUL56 as the primary target of letermovir, direct structural information regarding drug binding sites and conformational effects is still lacking (Ligat et al., 2018). This lack of direct evidence is a critical limitation; without a co-crystal or cryo-EM structure, computational docking and mutagenesis studies, while informative, cannot definitively distinguish between direct steric hindrance and allosteric perturbation of the terminase's mechanical cycle. As a result, several mechanistic hypotheses have been proposed (Ligat et al., 2019; Kmetsch et al., 2025).

This review focuses specifically on the molecular interaction between letermovir and the HCMV terminase complex. We have summarized current understandings of terminase architecture and function, discussed proposed mechanisms of inhibitor action, analyzed the structural implications of resistance mutations, and highlighted future directions for structure-guided development of next-generation terminase inhibitors.

## 2 Organization and function of the HCMV terminase complex

Genome packaging is an essential step during herpesvirus maturation (Kornfeind and Visalli, 2018; Li and Yu, 2020; Iwaisako and Fujimuro, 2024). Following viral DNA replication, long concatemeric DNA molecules should be cleaved into unit-length genomes and packaged into preassembled capsids. This process requires coordinated activity of the viral terminase machinery. The HCMV terminase complex is primarily composed of pUL56, pUL89, and pUL51. These proteins cooperate to recognize packaging signals, cleave concatemeric DNA, and translocate viral genomes into capsids. In the absence of experimentally resolved structures for the HCMV terminase complex, AlphaFold 3 (Abramson et al., 2024) was employed to predict the structures of pUL56 (Uniprot Code: P16724), pUL89 (Uniprot Code: P16732), pUL51 (Uniprot Code: P16792), and the trimer architecture (Figures 1A–E). pUL56, the largest protein in this trimer, contains Spool, Fix, Strut, Reinforce, Regulator, and Packaging domains (Figures 1A, B). pUL89 contains N-Lasso, Strut, ATPase, Regulator, and Nuclease domains (Figures 1A, C). pUL51 is the smallest one, containing only N-Lasso and Fix domains (Figures 1A, D). Critical regions responsible for normal function (including the Spool and packaging domains of pUL56, nuclease domain of pUL89) and trimer formation (including N-Lasso domains of pUL51 and pUL89, and ATPase domain of pUL89) display high prediction precision (Figures 1A–D, Supplementary Figures S1 A–C). The predicted models also show similarity to corresponding HSV-1 subunits, especially for pUL56 and pUL51 (Supplementary Table S1).

**Figure 1:**
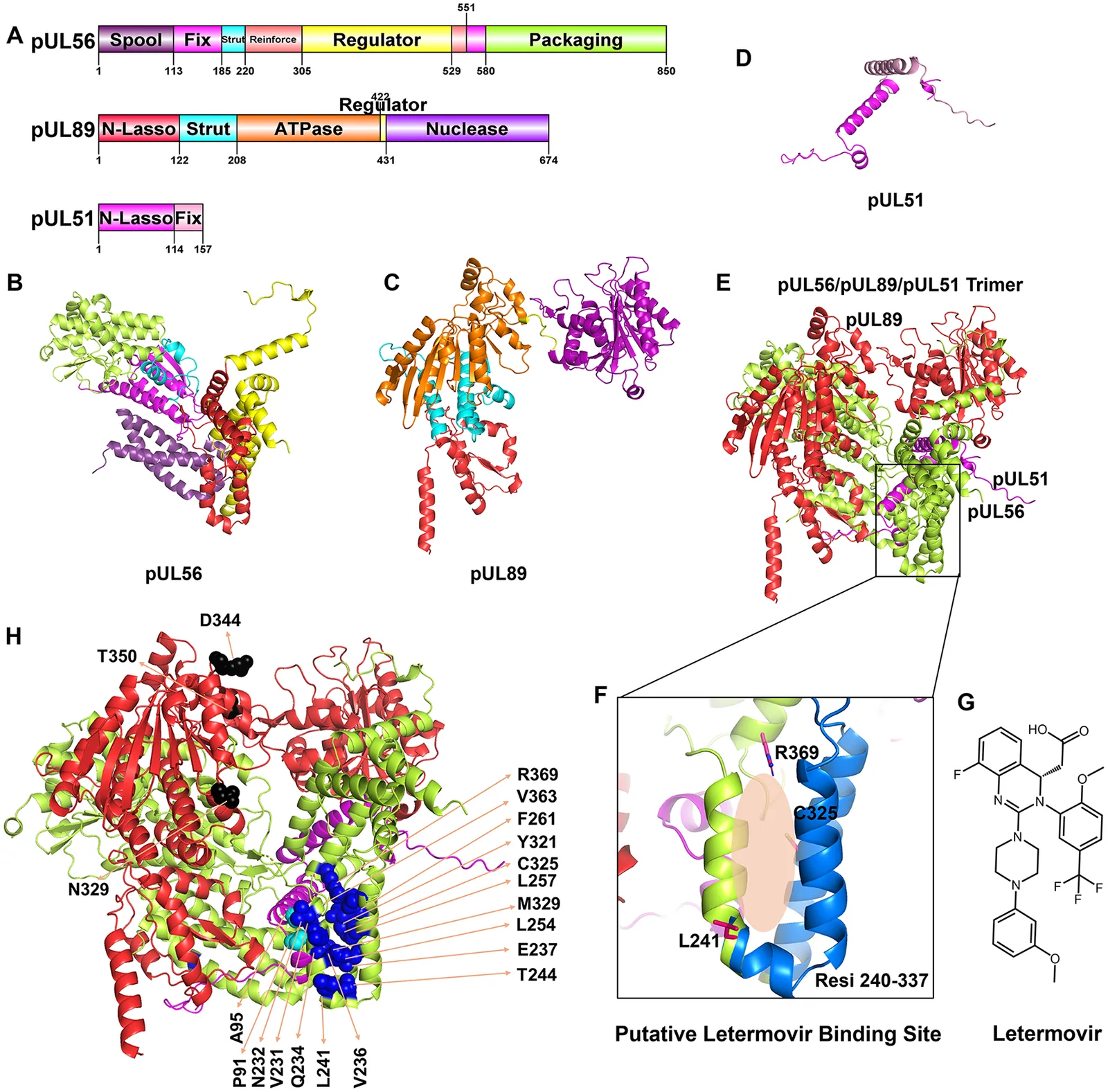
AlphaFold-predicted structural model of HCMV terminase and resistance mutations. **(A)** Domain structures of pUL56, pUL89, and pUL51. The domain designations follow the HSV-1 terminase structure (Yang et al., 2020). **(B–D)** Structural models of pUL56 **(B)**, pUL89 **(C)**, and pUL51 **(D)** by AlphaFold3. The regions are colored according to **(A)**. **(E)** Trimeric model of HCMV terminase by AlphaFold3. pUL56, green; pUL89, red; pUL51, magenta. **(F)** Putative letermovir binding site in pUL56. AlphaFold3-predicted model of HCMV terminase trimer is used for displaying putative letermovir binding site. Resistance mutation sites are shown as sticks. The region containing residues 240–337 is shown in blue. The putative binding site manifests as a binding pocket. **(G)** Molecular structure of Letermovir. **(H)** Resistance mutations in HCMV terminase. Residues with mutations of resistance index value greater than two are labeled. The mutational residues are shown as sphere and blue in pUL56, black in pUL89, and cyan in pUL51.

### 2.1 pUL56

pUL56 is widely recognized as the core regulatory subunit of the HCMV terminase complex and possesses both ATPase and nuclease activities (Hwang and Bogner, 2002; Scheffczik et al., 2002; Scholz et al., 2003; Iwaisako and Fujimuro, 2024). Although two ATP-binding sites have been identified in pUL56 suggesting that ATP-dependent conformational changes may facilitate genome packaging, only one of these sites is required for its enzymatic activity (Scholz et al., 2003). Critically, the functional significance of the second ATPase site remains unclear; it may represent a vestigial domain, a regulatory element that fine-tunes motor activity under specific conditions, or an artifact of *in vitro* assays that does not recapitulate the native packaging milieu. In addition, a LAGLIDADG homing endonuclease motif and a zinc finger motif are critical for pUL56 function and viral replication (Ligat et al., 2019). While the complete structure of full-length pUL56 remains experimentally undetermined, computational predictions support the presence of these essential sites and motifs. pUL56 is also proposed to function in viral DNA recognition by binding to AT-rich sequences, including pac 1 and pac 2 (Bogner et al., 1998). Complex formation requires only the AT-rich core sequences within these cis-acting motifs. Furthermore, direct physical interaction between pUL56 and the HCMV portal protein pUL104 suggests the essential role of pUL56 in portal docking and DNA packaging (Dittmer et al., 2005).

### 2.2 pUL89

Similar to pUL56, pUL89 also exhibits endonuclease activity (Scheffczik et al., 2002). Structural and biochemical analyses have shown that pUL89 harbors an endonuclease domain at its C-terminus whose structure was solved by X-ray crystallography, which is related to RNase H-like endonucleases (Nadal et al., 2010; Theiß et al., 2019). The enzymatic activity of pUL89 requires metal ions such as manganese. pUL89 is considered essential for HCMV, as inhibitors targeting its endonuclease domain can suppress HCMV replication and genome cleavage (Wang et al., 2017; Bongarzone et al., 2018; He et al., 2022; Jagtap et al., 2023). Compounds with metal-binding scaffolds, such as diketoacid derivatives and 4,5-dihydroxypyrimidine methyl derivatives, can inhibit the nuclease function of pUL89 and have demonstrated potential potency against HCMV. A significant caveat here is that the *in vitro* nuclease activity of pUL89, while well-documented, does not necessarily equate to its predominant role in the context of the full terminase complex; the relative contribution of pUL89 nuclease activity vs. that of pUL56 in the actual cleavage of concatemeric DNA during infection remains to be definitively dissected.

### 2.3 pUL51

pUL51 is the least characterized component of the terminase complex. Available evidence suggests that upon knockdown of UL51, neither unit-length genomes nor DNA-filled capsids can form, indicating that pUL51 plays a role in DNA packaging (Borst et al., 2013). Furthermore, UL51 is required for the subnuclear localization of pUL56 and pUL89. The interaction among all three components is essential for terminase complex formation, as the absence of any one component leads to a marked reduction in the other two (Neuber et al., 2017). This mutual dependence, however, complicates interpretation: it remains unclear whether pUL51 has a direct catalytic or mechanical function in packaging, or whether its primary role is as a scaffold or chaperone that stabilizes the complex and facilitates its nuclear import. Additionally, the C-terminal region of pUL51 is critical for terminase complex assembly, as mutations in this region are incompatible with viral growth (Neuber et al., 2018).

### 2.4 Mutual interaction between terminase subunits

The components of the HCMV terminase complex exhibit mutual dependence for proper assembly into a functional complex and for nuclear localization (Borst et al., 2013; Neuber et al., 2017). For example, pUL56 and pUL89 are not detectable in replication compartments when UL51 is knocked down (Borst et al., 2013). Furthermore, in the absence of UL51, no unit-length genomes are detected. Deletion of any terminase ORF from the HCMV genome markedly reduces the levels of the remaining terminase subunits, relative to cells transfected with the wild-type genome (Neuber et al., 2017). Additionally, the nuclear translocation of UL51 and UL89 requires all three subunits. Studies have shown that residues 580–600 of pUL89 are responsible for interaction with pUL56, while residues 514–522 and 671–680 of pUL56 mediate interaction with pUL89 (Thoma et al., 2006; Ligat et al., 2017; Gourin et al., 2025b; Mafi et al., 2025). Moreover, residues 671–680 and 757–764 contribute to stabilization of the ring-shaped structure by mediating contacts with pUL89 and pUL51 (Gourin et al., 2025b).

To date, herpesvirus packaging motors have only been characterized in HSV-1. Therefore, we constructed a structural model of the HCMV terminase complex using AlphaFold3 (Figure 1E). This model of HCMV terminase trimer displays a “V”-shaped structure reminiscent of the architecture of HSV-1 terminase trimer, and suggests extensive mutual interactions among pUL56, pUL89, and pUL51 (Figure 1E). Consistent with prior biochemical reports, our model recapitulates the interaction between pUL56 residues 514–522 and pUL89. In contrast, and notably, previously reported interfaces—namely pUL89 residues 580–600 and pUL56 residues 671–680—do not contribute to trimer formation in our prediction. This divergence is striking, as it suggests either that HCMV and HSV-1 terminases may adopt genuinely different quaternary arrangements, or that the prediction fails to capture conformation-dependent interfaces that are stabilized only under specific states during the packaging cycle. Rather than interpreting this discrepancy as evidence for or against similarity to HSV-1, we emphasize that these differences highlight the limitations of static computational models and the critical need for experimental structural validation (e.g., cryo-EM) of the HCMV complex. Until such data are available, the functional relevance of these predicted interactions—and their potential roles in genome packaging—remains speculative and requires cautious interpretation.

## 3 Letermovir and its interaction with HCMV terminase

The development of letermovir marked a major milestone in antiviral therapy because it introduced the first clinically approved inhibitor specifically targeting herpesvirus genome packaging (Lischka et al., 2010; Goldner et al., 2011). Letermovir exhibits potent anti-HCMV activity at nanomolar concentrations and demonstrates remarkable selectivity for HCMV relative to other herpesviruses. Importantly, its mechanism of action differs completely from classical polymerase inhibitors, allowing effective suppression of strains resistant to other approved antivirals (Lischka et al., 2010). Clinical studies demonstrated that letermovir significantly reduced HCMV infection and disease in hematopoietic stem cell transplant recipients (Marty et al., 2017). Moreover, the drug displays substantially improved safety profiles compared with traditional polymerase inhibitors (Moore et al., 2023; Usman et al., 2026).

Several properties make the terminase complex particularly attractive as an antiviral target (Goldner et al., 2011; Iwaisako and Fujimuro, 2024). First, terminase proteins are highly virus-specific and lack direct human homologs. Second, genome packaging represents an essential late-stage step during viral replication. Inhibition of packaging prevents production of infectious virions even when viral DNA replication occurs (Wang et al., 2017). One of the most important unresolved questions in the field concerns how letermovir interacts with the terminase machinery to inhibit viral genome packaging. Despite these advances and advantages, the molecular mechanism underlying letermovir inhibition remains incompletely understood.

Several observations support the central importance of pUL56 in antiviral inhibition, because the majority of clinically observed letermovir resistance mutations map to pUL56, rarely to pUL89 and pUL51 (Goldner et al., 2011; Chou, 2015, 2017b; Bravo et al., 2022; Grgic and Gorenec, 2024; Gourin et al., 2025a). pUL56 was initially identified as the primary target of letermovir by generating and mapping drug-resistant HCMV mutants (Goldner et al., 2011). Key resistance mutations, including L241P and R369S, were localized to the UL56 gene (Figure 1F), and the drug was shown to inhibit viral DNA cleavage and packaging without affecting DNA replication.

A putative binding pocket within pUL56 has been identified through *in silico* docking and homology modeling, which is possibly located in the region residues 240–337 (Kmetsch et al., 2025, Figure 1F). It was found that in the presence of the most frequent mutations under high letermovir treatment, C325F and C325Y, letermovir might be pushed out of the binding pocket (Figures 1F–G). Consistently, the putative binding site of letermovir has a pocket which may accommodate the molecule (Figures 1F–G). Other study used NanoBiT-based assays to show that letermovir, even at 10 × its IC_50_, does not directly disrupt pUL56-pUL89 or pUL56-pUL51 interactions (Gourin et al., 2025a). Thus, the drug does not act as a simple interaction blocker. Instead, resistance mutations differentially modulate subunit binding—V236M enhances pUL56-pUL89 association while C325Y reduces it—suggesting that letermovir likely interferes with conformational dynamics (Gourin et al., 2025a).

## 4 Resistance mutations and their structural implications

The emergence of antiviral resistance has yielded important insights into the interaction between letermovir and the terminase complex. Critically, these resistance mutation strains are not merely laboratory artifacts but are bona fide clinical entities that arise under drug selection pressure in immunocompromised patients, particularly hematopoietic stem cell and solid organ transplant recipients, where prolonged prophylaxis or treatment provides the selective environment for resistance to emerge (Zhu et al., 2024). As summarized in Table 1, the majority of clinically relevant resistance mutations map to pUL56. These mutations are not evenly distributed across the gene but rather cluster in specific regions, distinct from a conventional catalytic site. Their locations are illustrated in Figure 1H for visual reference. Notably, most pUL56 resistance mutations are situated near the putative letermovir binding site with moderate prediction precision (Supplementary Figure S1D), where they may directly modify the drug-binding pocket and impair letermovir binding, thus conferring drug resistance (Figure 1H). Commonly implicated residues include V231, V236, L241, C325, and R369 (Goldner et al., 2014; Chou and Kleiboeker, 2022; Perchetti et al., 2023). Among these, mutations at C325 are particularly well characterized and are frequently associated with high-level resistance (Goldner et al., 2014; Chou, 2015; Chou and Kleiboeker, 2022). V231, C325 and R369 are located at the putative letermovir binding site (Figure 1H), whose mutation can impact letermovir binding directly. However, it is important to critically evaluate the assumption that these mutations confer resistance solely through reduced drug binding. An alternative, and equally plausible, explanation is that these mutations shift the conformational equilibrium of the terminase toward a state that is refractory to inhibition, without necessarily altering the affinity of letermovir for the isolated subunit. Distinguishing between these mechanisms requires direct biophysical binding measurements, which are currently lacking.

**Table 1:** Resistance mutations and their locations.

Mutations	Location	RI^a^	Reference
L241P	pUL56 Reinforce	220	Goldner et al. (2011)^b^
R369S	pUL56 Regulator	65	
C325Y	pUL56 Regulator	5870	Goldner et al. (2014)^b^
L241P	pUL56 Reinforce	268	
R369S	pUL56 Regulator	81	
V231L	pUL56 Reinforce	28	
R369M	pUL56 Regulator	23	
V236M	pUL56 Reinforce	19	
R369G	pUL56 Regulator	13	
T350M	pUL89 ATPase	2.8	Chou (2017b)
N329S	pUL89 ATPase	2.0	
D344E	pUL89 ATPase	1.8	
N232Y	pUL56 Reinforce	17	
E237D	pUL56 Reinforce	16	
M329T	pUL56 Regulator	4.5	
P91S	pUL51 N-Lasso	2.1	Chou (2017a)
L241P	pUL56 Reinforce	173	
V236M	pUL56 Reinforce	32	
R369M	pUL56 Regulator	16	
L257F	pUL56 Reinforce	8.6	
V231L	pUL56 Reinforce	5.6	
L254F	pUL56 Reinforce	3.2	
C325F	pUL56 Regulator	>3000	Chou (2015)
V236M/L257I/M329T	pUL56 Reinforce/Regulator	>3000	
C325R	pUL56 Regulator	>3000	
V236L/L257I	pUL56 Reinforce	260	
E237D/T244K/F261L	pUL56 Reinforce	104	
V236L	pUL56 Reinforce	14	
E237D	pUL56 Reinforce	10	
T244K/F261L	pUL56 Reinforce	8.2	
V231L	pUL56 Reinforce	5.1	
L257I	pUL56 Reinforce	4.9	
Y321C	pUL56 Regulator	4.6	
F261C	pUL56 Reinforce	4.4	
M329T	pUL56 Regulator	4.4	
T244K	pUL56 Reinforce	3.3	
F261L	pUL56 Reinforce	2.8	
C325Y	pUL56 Regulator	>8000	Chou and Kleiboeker (2022)
C325W	pUL56 Regulator	>8000	
C325R	pUL56 Regulator	>3000	
C325F	pUL56 Regulator	>3000	
R369T	pUL56 Regulator	52	
R369G	pUL56 Regulator	44	
R369S	pUL56 Regulator	38–48	
V236M	pUL56 Reinforce	32–50	
N232Y	pUL56 Reinforce	17	
L257F	pUL56 Reinforce	9–14	
V231L	pUL56 Reinforce	5–8	
R369K	pUL56 Regulator	4.9	
V363I	pUL56 Regulator	3.7	
Q234R	pUL56 Reinforce	2.0	
C325Y	pUL56	-	Perchetti et al. (2023)
A95V	pUL51	13.8	Muller et al. (2022)

^a^RI, resistance index; the EC_50_ for the mutant divided by the EC_50_ for the wild-type. ^b^Results from letermovir-resistant HCMV mutants obtained *in vitro*.

Compared with pUL56, resistance mutations in pUL51 and pUL89 are less frequently observed. Within pUL51, two resistance-associated residues—P91 and A95—have been identified (Muller et al., 2022). These residues are positioned near the putative letermovir binding site but lie outside the binding pocket itself (Figure 1H). As such, these mutations may induce local conformational changes that indirectly hinder inhibitor recognition. Nevertheless, P91S does not significantly impact resistance, unlike A95V (Chou, 2017a; Muller et al., 2022). In contrast, resistance residues in pUL89—including N329, D344, and T350—are situated at a considerable distance from the putative binding site (Chou, 2017b). Rather than directly affecting drug binding, these mutations may modulate the global conformational dynamics of the terminase complex, thereby circumventing inhibition. These mutations only modestly induce resistance. Such distal effects likely reflect coordinated conformational transitions that are integral to the genome packaging process. The modest resistance indices associated with pUL89 and pUL51 mutations raise an important caveat: these variants may not represent true clinical resistance but rather compensatory mutations that arise under selective pressure to restore fitness, or they may be polymorphisms that have been statistically associated with letermovir exposure without a direct causal role in drug evasion. The threshold for what constitutes clinically meaningful resistance (as opposed to *in vitro* reduced susceptibility) remains a subject of ongoing debate.

## 5 Future directions in terminase-targeted antiviral development

Currently, no high-resolution structure of the intact HCMV terminase complex has been determined. In particular, structures of drug-bound terminase assemblies are lacking. This absence represents a major obstacle to fully understand antiviral inhibition and subsequent antiviral development. Recent technological advances are beginning to address these limitations. Cryo-electron microscopy has revolutionized structural virology and may enable visualization of intact packaging intermediates (Yang et al., 2020; Chechik et al., 2024; Xu et al., 2026). Improvements in detector technology and image processing now permit analysis of increasingly dynamic macromolecular assemblies (Amann et al., 2023; Shahid et al., 2025; Yi et al., 2026). Artificial intelligence-based structure prediction tools such as AlphaFold have also accelerated structural modeling of viral proteins (Jumper et al., 2021; Abramson et al., 2024). Although predicted structures require experimental validation, they may provide valuable frameworks for interpreting resistance mutations and guiding mechanistic hypotheses. A critical consideration for future structural efforts is that the terminase might be a highly dynamic molecular motor. Obtaining a static structure, while invaluable, may not reveal the mechanistically relevant conformations that exist transiently during ATP hydrolysis and DNA translocation. Time-resolved cryo-EM or techniques that trap the complex in specific nucleotide-bound states might be essential to understand the full conformational cycle and how letermovir perturbs it.

The success of letermovir has stimulated intense interest in developing next-generation terminase inhibitors (Ourahmane et al., 2026). One major goal involves identifying compounds capable of overcoming existing resistance mutations. Structure-guided drug design may enable development of inhibitors targeting alternative conformational states or distinct interaction surfaces (Kmetsch et al., 2025; Mafi et al., 2025). Broad-spectrum herpesvirus terminase inhibitors also represent an attractive possibility (Dehghanpir et al., 2018). Although letermovir displays remarkable specificity for HCMV, conserved mechanistic principles among herpesvirus packaging systems suggest that broader antiviral targeting may be achievable (Andronova et al., 2024; Iwaisako and Fujimuro, 2024). Encouragingly, this concept has recently been validated with the description of LN-7, a baloxavir-inspired inhibitor targeting the pUL15 nuclease domain of the HSV terminase complex (Andreu et al., 2025). LN-7 has demonstrated potent antiviral activity in both cell culture and a murine model, and notably retained efficacy against acyclovir-resistant clinical strains. However, the high specificity of letermovir for HCMV, while advantageous for safety, also highlights the challenge of developing broad-spectrum inhibitors: the sequence and structural divergence among herpesvirus terminases may preclude a single compound from achieving potent inhibition across multiple viruses without extensive optimization. The trade-off between breadth of activity and selectivity should be carefully considered in any development program.

Combination therapies may provide another important strategy. Simultaneous inhibition of viral DNA replication and genome packaging could reduce resistance emergence and improve antiviral efficacy (Wildum et al., 2015; O'Brien et al., 2018). Future inhibitors may additionally exploit allosteric regulatory mechanisms. Rather than targeting catalytic residues directly, compounds that perturb conformational coordination within the packaging motor may achieve potent antiviral activity (Thaljeh et al., 2019). Allosteric inhibitors may offer the advantage of targeting less conserved, yet functionally critical, interfaces that may have a higher barrier to resistance development (Gable et al., 2014; Chen et al., 2017), but they also require a detailed understanding of the dynamic conformational landscape that is currently lacking. Targeted protein degradation strategies such as PROTAC-based approaches could also represent promising future directions (Salimova et al., 2025; Zhou et al., 2025). Degradation of essential packaging components may provide advantages over conventional occupancy-driven inhibition. While conceptually appealing, PROTAC approaches face significant hurdles for antiviral development, including the need for cell-permeable, orally bioavailable molecules that can engage the viral protein and the host E3 ligase machinery simultaneously, as well as the potential for off-target degradation of host proteins (Jin et al., 2025; Zhou et al., 2026).

Ultimately, progress in this field will depend heavily on improved structural understanding of terminase architecture and dynamics. A crucial, and often underappreciated, bottleneck is the difficulty of expressing and purifying sufficient quantities of the intact, functional HCMV terminase complex for biochemical and structural studies. Investment in optimizing expression systems and reconstitution protocols is a prerequisite for the structural advances that will underpin rational drug design.

## 6 Conclusion

In summary, the HCMV terminase complex is a validated antiviral target, yet a critical gap persists between correlative evidence—resistance mutations, computational docking, and cellular assays—and a truly mechanistic understanding of letermovir inhibition. Direct structural and biophysical data on drug binding are urgently needed, as current inferences remain speculative. The field should move from genetics to biochemistry—through high-resolution structures, binding measurements, and reconstituted packaging assays—to resolve whether letermovir traps a conformational state or kinetically disrupts motor function, and to enable rational design of next-generation inhibitors with higher resistance barriers.
