# Supplementary material for: The HCMV terminase as an antiviral target: structural insights, inhibitory mechanisms, and the era of letermovir
Source: Front Microbiol. 2026 Sep 18;17:1942629. doi: 10.3389/fmicb.2026.1942629 (PMC13633068; doi:10.3389/fmicb.2026.1942629)
Supplement: Supplementary file 1 [file Supplementary_file_1.docx]

**Figure S1 Prediction results for HCMV terminase**. (A-D) The prediction results using AlphaFold 3 for pUL56 (A), pUL89 (B), pUL51 (C), and terminase trimer (D). plDDT, pTM and ipTM scores are provided. The dashed circle in D indicates the putative letermovir binding site.

**Table S1 Comparison of predicted models of HCMV terminase with available experimental structures of HSV-1 terminase.**

| **HCMV subunit-HSV-1 subunit** | **RMSD (Å)** |
| --- | --- |
| pUL56-pUL28 | 1.7 (for 431 Cα atom) |
| pUL51-pUL33 | 0.6 (for 60 Cα atom) |
| pUL89-pUL15 | 4.3 (for 536 Cα atom) |
